# A new method to recover L-tyrosine from *E. coli* fermentation broth

**DOI:** 10.1080/21655979.2020.1827893

**Published:** 2020-10-23

**Authors:** Guohua Li, Jingyang Liu, Ning Chen, Qingyang Xu

**Affiliations:** a Biological Engineering, Tianjin University of Science and Technology, Tianjin, China; b Department of Bioengineering, Tianjin University of Science and Technology, Tianjin, China

**Keywords:** L-tyrosine, fermentation broth, recovery, cell lysis, acidification

## Abstract

Although the production of L-tyrosine by recombinant *Escherichia coli* has been widely reported, L-tyrosine recovery from the fermentation broth has been rarely reported. Methods to recover L-tyrosine from the broth after alkaline lysis of the bacterial cells have been described. However, the broth becomes viscous and dark following cell lysis, making further extraction and purification difficult. Here, a new method for L-tyrosine extraction and purification from the fermentation broth without the lysis of bacteria is reported. First, acids, rather than bases, were used to dissolve L-tyrosine in the broth without causing lysis of the bacterial cells. *E. coli* cells in the broth were then removed through centrifugation. Activated carbon was then used to decolorize the supernatant containing L-tyrosine. Finally, sodium hydroxide was added to the clarified L-tyrosine solution for isoelectrocrystallization. L-tyrosine was obtained after filtration and drying. The recovery yield of L-tyrosine was 92%, and the purity was >98.5%, indicating high efficiency of the new method of L-tyrosine recovery from fermented broth. Furthermore, the method provides a reference for the extraction of guanosine, inosine, hypoxanthine, and other biological fermentation products.

## 1. Introduction

L-tyrosine is commonly used as an additive and nutritional supplement in the food industry; additionally, it has important applications in the medical and chemical industries [1,2]. In recent years, with the development of synthetic biology, L-tyrosine production by microbial fermentation has gained great research interest. Ranjan et al. [3] used modified *Escherichia coli*, which produced 55 g/L of L-tyrosine in a 200 L fermentation tank after 48 h. Byoungjin et al. [4] reported that IPTG-induced *E. coli* BTY2.13 could produce 43.14 g/L of L-tyrosine through fed-batch fermentation. Xu et al. [5] reported a heat-induced L-tyrosine-producing strain (*E. coli* HRP) was used to ferment 55.54 g/L of L-tyrosine in a 3 L fermentation tank through fed-batch fermentation. Our team also developed an L-tyrosine-producing strain, *E. coli* GHLTYR-168, which could ferment 42.2 g/L L-tyrosine by batch feeding in a 3 L bioreactor for 24 h [6]. However, L-tyrosine recovery from fermentation broth is rarely reported. To the best of our knowledge, only Ranjan et al. have reported two methods of L-tyrosine extraction from *E. coli* fermentation broth. One method involves centrifugation of L-tyrosine crystals from the biomass, based on their different densities. In practice, however, L-tyrosine recovery and cell removal rates are difficult to balance to achieve a good separation effect. In another method, L-tyrosine is first dissolved at pH 11.5, followed by acid precipitation from clarified supernatants after removal of the biomass through membrane filtration. However, the fermentation broth has a high viscosity and darkens at pH >9.0, because. following cell lysis after adding alkali to the fermented broth, the cell contents spill out, and the proteins are denatured. The filtration or centrifugation of the fermentation broth, for removal of the biomass, becomes difficult. Therefore, it is necessary to develop new and efficient methods to recover L-tyrosine from *E. coli* fermentation broth without lysis of the cells.

In this study, a new method to recover L-tyrosine from *E. coli* fermentation broth was developed. Acids were added to the fermentation broth to adjust the pH to 0.5 to dissolve the L-tyrosine crystals, while the *E. coli* cells were not lysed. The *E. coli* cells in the fermentation broth were then removed by centrifugation. Thereafter, L- tyrosine was obtained by decolorization, crystallization, and drying.

## 2. Materials and methods

### 2.1. Strains and media

*E. coli* GHLTYR-168 was provided by the Tianjin University of Science and Metabolic Engineering Laboratory. The genetic background and culture methods of this strain have been previously reported [6]. This strain can produce substantial amounts of L-tyrosine through fed-batch fermentation.

### 2.2. Analysis method

Biomass was expressed in terms of cell dry weight (cell dry weight = 0.364 × OD_600_), where OD_600_ refers to the absorbance of the fermentation broth measured at 600 nm, as determined using a spectrophotometer [7]. Glucose in the fermented broth was measured using a bioanalyzer [8], and amino acid concentrations were measured using an automatic amino acid analyzer [7]. The acetic acid in the fermented broth was measured using high-performance liquid chromatography [5]. The fermented broth was centrifuged at 30 degree Celsius using a high-speed centrifuge (CR-22GIII, Hitachi, Japan). L-tyrosine crystals and cell morphology in the fermentation broth were observed via microscopy (BH2, Olympus, Japan). The quality of L-tyrosine obtained from the fermentation broth was tested according to the USP32 standard and method [https://www.drugfuture.com/Pharmacopoeia/usp32/pub/data/v32270/usp32nf27s0_m87220.html].

## 3. Results and discussion

### 3.1. Composition analysis of the fermentation broth

It is necessary to select a suitable strategy to recover L-tyrosine, according to the characteristics of the fermentation broth [9]. The fermentation broth is mainly divided into solid and liquid phases but is not stratified. The main components of the solid phase are *E. coli* cells and L-tyrosine crystals. In the fermentation broth, the biomass of *E. coli* was 43.8 g/L, and the L-tyrosine titer was 42.2 g/L. In addition, wastewater (liquid phase) occupies majority of the fermentation broth and contains a large number of soluble components. These soluble components include both unused substrates and byproducts of cell metabolism. Table 1 shows the composition of the wastewater (supernatant of fermentation broth). Because the solubility of L-tyrosine is extremely low, it mostly exists in the crystal form. Thus, the fermentation broth displays a silvery color of L-tyrosine. The amount of L-tyrosine dissolved in wastewater was very small, i.e., 0.729 g/L. In addition, the amino acid analyzer detected several other amino acids, such as leucine, aspartic acid, and arginine; the concentrations of these amino acids were <0.4 g/L.

**Table 1.** Ingredients in the supernatant of fermentation broth.

Composition	Concentration (g/L)	Composition	Concentration (g/L)
L-tyrosine	0.729 ± 0.021	Valine	0.090 ± 0.019
Acetic acid	0.413 ± 0.155	Alanine	0.057 ± 0.011
Leucine	0.342 ± 0.053	Serine	0.029 ± 0.009
Aspartic acid	0.217 ± 0.041	Threonine	0.021 ± 0.008
Lysine	0.211 ± 0.014	Isoleucine	0.021 ± 0.011
Arginine	0.132 ± 0.011	Histidine	0.020 ± 0.021
Phenylalanine	0.125 ± 0.021	Glutamate	0.016 ± 0.004

The data in the table is the average of the three experiments.

### 3.2. L-tyrosine recovery from the fermentation broth

#### 3.2.1. Removal of E. coli cells from the fermentation broth

Sulfuric acid (98%, m/m) was added to the fermentation broth until the pH value reached 0.5. At this point, the L-tyrosine crystals were completely dissolved in the liquid phase. Concurrently, *E. coli* cells in the fermentation broth did not lyse, as observed under a microscope. Moreover, the color and viscosity of the fermentation broth did not change because the contents of the cells were not released. Hydrochloric acid also achieves the same effect; however, chlorine ions may aggravate the corrosion of equipment and thus were not used [10].

Although the addition of alkali can also dissolve L-tyrosine [3], the *E. coli* cells are lysed, after which a large number of cell fragments, nucleic acids, and proteins are released [11]. Following are few drawbacks of cell lysis: (1) The broth becomes very viscous; thick fermented broth, like the thick mucus of a flu patient, is very difficult to handle later. (2) The soluble components (proteins and pigments) of the cell enter the fermentation broth, introducing additional impurities; removing these impurities increases the difficulty and overall cost and is one of the significant technical challenges in the downstream purification and separation process from the laboratory to the plant.

The same challenge also arises in the production of recombinant proteins, enzymes, and plasmids through high-density fermentation using *E. coli* [12]. Therefore, dissolving L-tyrosine is the best method to recover L-tyrosine from *E. coli* fermentation broth without lysing the bacteria. In addition, we have wrongly suspected that high biomass density is the cause of the increased viscosity of the fermented broth after alkali addition. The biomass of *E. coli* GHLTYR-168 in this study was 43.8 g/L while that of *E. coli* K12 [3] was only 23.4 g/L. However, when sodium hydroxide was added to *E. coli* GHLTYR-168 fermentation broth with only 20 g/L biomass, the viscosity remained high. Another method to recover L-tyrosine from *E. coli* fermentation broth has been reported [3]. The densities of L- tyrosine and the cells are different. Theoretically, they can be separated through centrifugation. However, practically, the effect is not ideal. A considerable number of L-tyrosine crystals and *E. coli* cells are bound together, and the centrifuge cannot separate them. The same phenomenon has been reported previously [3]. Therefore, biomass is not the cause of viscosity of the fermentation broth after alkali addition.

The broth was then centrifuged at 8,000 rpm for 10 min. *E. coli* cells were deposited at the bottom of the centrifuge tube. Approximately 240 g of wet *E. coli* cells can be collected per liter of fermentation broth. Most L-tyrosine was dissolved in a transparent pale yellow supernatant. Thereafter, the cells at the bottom of the centrifuge cup could be washed, and the second supernatant after centrifugation merged with the first supernatant. In addition to centrifugation, ceramic membrane filtration could also remove *E. coli* cells. However, almost all ceramic membrane filtration equipment and industrial centrifuges are supported by stainless steel. The extraction of L-tyrosine in a highly acidic environment accelerates corrosion and damage, which has caused concern in the industry.

#### 3.2.2. Decolorization

The supernatant was poured into a beaker and decolorized with 0.5% (m/V) wood powder activated carbon (Jibao, Shanghai, China). The beaker was heated in a water bath to control the temperature at 80°C for 20 min. The solution in the beaker was then filtered to remove the activated carbon. The filtration proceeds through two steps: (1) filter paper (aperture 30 µm) filtration and (2) microporous membrane (aperture 0.22 micron) filtration. In addition, the filter paper and microporous membrane should be coated with diatomite, approximately 0.5 cm thick, before filtering, to improve filtration performance and reduce activated carbon from plugging the filtration medium [13,14]. A decolorized solution (colorless and transparent) containing L-tyrosine was obtained.

#### 3.2.3. Crystallization and drying

The L-tyrosine decolorization solution was cooled to below 10°C. Thereafter, the decolorizing fluid was adjusted to pH 5.7 with sodium hydroxide. A large number of white flocculent or needlelike crystals appeared in the solution that were identified as L-tyrosine. Wet L-tyrosine crystals were filtered using microporous membranes and then collected. Wet L-tyrosine crystals were dried to a constant weight of 80°C in an oven, and dry L-tyrosine was obtained. To further improve the purity of L-tyrosine, recrystallization or cold water washing could be used. The average extraction yield was 92%.

### 3.3. Analysis of L-tyrosine

The purity of L-tyrosine extracted from the fermentation broth was greater than 98.5%. L-tyrosine was obtained as a white crystalline powder. The specific rotation was between −9.5° and −10.8°, calculated using an automatic polarimeter (WZZ-2S, INESA, Shanghai, China). The transmittance was higher than 98.5%. These parameters indicate that L-tyrosine recovered by the method described above meets industrial-grade strands.

To our knowledge, the methods described in this paper are comparable in terms of yield and purity. The novelty of the method of L-tyrosine extraction and purification from *E. coli* fermentation broth introduced in this paper lies in the prevention of lysis of bacterial cells. This has two advantages: (1) the viscosity of the fermentation broth does not change, and (2) substances in the bacteria are not released into the fermentation broth, reducing the cost of further removal of impurities. Indeed, this method has unique characteristics. The fermented products must be secreted extracellularly and should be insoluble or slightly soluble under neutral conditions but soluble under acidic conditions. This method can be used as a reference for fermentation of other biological products, such as guanosine, inosine, hypoxanthine, and tryptophan.

## 4. Conclusion

This study aimed to extract and purify L-tyrosine from fermented broth efficiently. L-tyrosine in the fermented broth was dissolved, centrifuged, decolorized, crystallized, and dried to achieve a purity of >98.5%. The yield of L-tyrosine recovered from *E. coli* broth averaged 92%. This is a new and efficient method to recover L-tyrosine from *E*. fermentation broth. The main advantage of this method is that it does not require lysis of cells, overcoming the disadvantage of the previous method involving alkaline lysis, which causes the broth to become thick and dark. It can provide a reference for the extraction of guanosine, inosine, hypoxanthine, and other biological fermentation products.

## References

[1] Zhou S, Zhou C, Lu Q, et al. Convenient access to L-3,4,5-trioxygenated phenylalanine compounds from L-tyrosine. Tetrahedron. 2020;76(24):1–5.

[2] Yao J, He Y, Su N, et al. Developing a highly efficient hydroxytyrosol whole-cell catalyst by de-bottlenecking rate-limiting steps. Nat Commun. 2020;11(1):1–12. 32251291 10.1038/s41467-020-14918-5 PMC7090077

[3] Patnaik R, Zolandz RR, Green DA, et al. L-tyrosine production by recombinant Escherichia coli: fermentation optimization and recovery. Biotechnol Bioeng. 2008;99(4):741–752. 18069696 10.1002/bit.21765

[4] Byoungjin K, Robert B, Uk KH, et al. Metabolic engineering of Escherichia coli for the enhanced production of L-tyrosine. Biotechnol Bioeng. 2018;38:745–765. 10.1002/bit.26797 30019750

[5] Xu S, Wang Q, Zeng W, et al. Construction of a heat-inducible Escherichia coli strain for efficient de novo biosynthesis of l-tyrosine. Process Biochem. 2020;92:85–92.

[6] Li G, Chen Z, Chen N, et al. Enhancing the efficiency of L-tyrosine by repeated batch fermentation. Bioengineered. 2020;11(1):852–861. 32749196 10.1080/21655979.2020.1804177 PMC8291887

[7] Wu H, Tian D, Fan X, et al. Highly efficient production of L-histidine from glucose by metabolically engineered Escherichia coli. ACS Synth Biol. 2020;9:1–30. 32470291 10.1021/acssynbio.0c00163

[8] Ma H, Fan X, Cai N, et al. Efficient fermentative production of l-theanine by Corynebacterium glutamicum. Appl Microbiol Biotechnol. 2019;104(1):119–130. 31776607 10.1007/s00253-019-10255-w

[9] Khunnonkwao P, Jantama K, Kanchanatawee S, et al. A two steps membrane process for the recovery of succinic acid from fermentation broth. Sep Purif Technol. 2018;207:451–460.

[10] Perren RA, Suter T, Solenthaler C, et al. Corrosion resistance of super duplex stainless steels in chloride ion containing environments: investigations by means of a new microelectrochemical method. Corros Sci. 2001;43(4):707–726.

[11] Kamat V, Pandey S, Paknikar K, et al. A facile one-step method for cell lysis and DNA extraction of waterborne pathogens using a microchip. Biosens Bioelectron. 2018;99:62–69. 28738229 10.1016/j.bios.2017.07.040

[12] El-Ashram S, Suo X. Nucleic acid protocols: extraction and optimization. Biotechnol Reports. 2016;12:33–39. 10.1016/j.btre.2016.10.001 PMC5361071 28352552

[13] Dan G, Hualin W, Pengbo F, et al. Diatomite precoat filtration for wastewater treatment: filtration performance and pollution mechanisms. Chem Eng Res Des. 2018;137:403–411.

[14] Ren Z, Gao H, Zhang H, et al. Effects of fluxes on the structure and filtration properties of diatomite filter aids. Int J Miner Process. 2014;130:28–33.

